# Fully automated analysis of cardiotoxicity surveillance echocardiograms: a direct test–retest precision comparison with experts

**DOI:** 10.1093/ehjimp/qyag142

**Published:** 2026-08-21

**Authors:** Benjamin Dowsing, Katia Menacho, Verónica Culotta, Benjamin Meredith, Beth Unsworth, Adam Ioannou, Anish Bhuva, Rhodri Davies, Ana Ferreira, Arjun Ghosh, Mark Westwood, Leon Menezes, Marianna Fontana, Guy Lloyd, Matthew Shun-Shin, James C Moon, Charlotte H Manisty

**Affiliations:** Bart’s Heart Centre, Saint Bartholomew’s Hospital, London, UK; Institute of Cardiovascular Science, University College London, Gower Street, London WC1E 6BT, UK; Bart’s Heart Centre, Saint Bartholomew’s Hospital, London, UK; Institute of Cardiovascular Science, University College London, Gower Street, London WC1E 6BT, UK; Bart’s Heart Centre, Saint Bartholomew’s Hospital, London, UK; Bart’s Heart Centre, Saint Bartholomew’s Hospital, London, UK; National Heart and Lung Institute, Imperial College London, London, UK; Deparment of Cardiology, Imperial College Healthcare NHS Trust, London, UK; Bart’s Heart Centre, Saint Bartholomew’s Hospital, London, UK; National Amyloidosis Centre, University College London, Royal Free Campus, London, UK; Bart’s Heart Centre, Saint Bartholomew’s Hospital, London, UK; Institute of Cardiovascular Science, University College London, Gower Street, London WC1E 6BT, UK; Bart’s Heart Centre, Saint Bartholomew’s Hospital, London, UK; Institute of Cardiovascular Science, University College London, Gower Street, London WC1E 6BT, UK; Bart’s Heart Centre, Saint Bartholomew’s Hospital, London, UK; Bart’s Heart Centre, Saint Bartholomew’s Hospital, London, UK; Institute of Cardiovascular Science, University College London, Gower Street, London WC1E 6BT, UK; Bart’s Heart Centre, Saint Bartholomew’s Hospital, London, UK; Bart’s Heart Centre, Saint Bartholomew’s Hospital, London, UK; National Amyloidosis Centre, University College London, Royal Free Campus, London, UK; Bart’s Heart Centre, Saint Bartholomew’s Hospital, London, UK; Institute of Cardiovascular Science, University College London, Gower Street, London WC1E 6BT, UK; National Heart and Lung Institute, Imperial College London, London, UK; Deparment of Cardiology, Imperial College Healthcare NHS Trust, London, UK; Bart’s Heart Centre, Saint Bartholomew’s Hospital, London, UK; Institute of Cardiovascular Science, University College London, Gower Street, London WC1E 6BT, UK; Bart’s Heart Centre, Saint Bartholomew’s Hospital, London, UK; Institute of Cardiovascular Science, University College London, Gower Street, London WC1E 6BT, UK

**Keywords:** echocardiography, diagnosis, imaging, innovation, screening

## Abstract

**Aims:**

Serial imaging demands precision—particularly for cardiotoxicity screening where small changes gatekeep major decisions. Automated measurement using artificial intelligence (AI)-based techniques should reduce variability and increase confidence for detection of true change. We directly compared the precision of fully automated AI vs. expert manual analysis of left ventricular function by echocardiography.

**Methods and results:**

Consecutive cancer patients referred for cardiotoxicity monitoring underwent same-day repeat 2D echocardiography [*n* = 60, 83% female, mean left ventricular ejection fraction (LVEF) 57 ± 7%], with 3D acquisition where feasible (*n* = 45). All 2D images underwent blinded analysis by a fully automated analysis software and four experts. Quantitative 2D LVEF measurement was feasible in 96% scans by both methods. Mean absolute difference (MAD) between repeat scans was significantly lower for AI than experts for both 2D LVEF {3.6% [confidence interval (CI) 2.8–4.4] vs. 6.6% [CI 5.3–7.9], *P* = 0.011} and global longitudinal strain [1.5% (CI 1.1–1.9) vs. 2.5% (CI 2.0–3.0), *P* = 0.006]. In participants with 3DE datasets, 3D LVEF MAD was lower than manual 2D LVEF [3.6% (CI 2.8–4.6) vs. 6.4% (CI 5.0–8.0), *P* = 0.006] but comparable to AI-derived 2D LVEF [3.5% (CI 2.9–4.2), *P* = 0.4]. In a subset (*n* = 48) also with same-day cardiovascular magnetic resonance (CMR) studies, AI 2DE analysis demonstrated stronger agreement with CMR than expert 2DE analysis [LVEF MAD 5.0% (CI 4.1–5.9) vs. 7.8% (CI 6.6–9.0) *P* = 0.006].

**Conclusion:**

In cancer patients at risk of cardiotoxicity, the precision of 2D echocardiographic AI analysis exceeds that of experts, matching that of 3D LVEF assessment.

**Trial Registration:**

NCT06817044

**Lay summary:**

Some cancer treatments may weaken the heart. As such, at-risk patients have repeat heart scans (echocardiograms) over the course of their cancer treatment. Doctors monitor these scans over time and watch closely for any change; even a small decline in heart function can trigger a major decision, such as pausing life-saving cancer therapy. It is therefore vital that these measurements are as consistent or ‘precise’ as possible.

The problem is that measurements taken by hand can vary widely, and ‘precision’ may be low, even when performed by skilled experts. This makes it hard to know whether a change in heart function across follow-up scans is real or not and whether any action is therefore needed. We tested whether artificial intelligence (AI) software could measure heart function more precisely than experts.

Sixty cancer patients each had two echocardiographic heart scans in close succession on the same day. This approach purposefully reduces the impact of other factors (such as differences in the person or equipment performing the heart scan) that might otherwise mean results differ. We then compared how closely the repeat measurements of heart function matched when performed by AI vs. four echocardiography experts. The AI was more precise, giving closer results between the two scans, whilst the experts’ results varied more.

In short, AI can measure heart function more precisely than experts in patients with cancer. This could give doctors greater confidence that any change seen on a scan is genuine, helping them make safer, better-informed decisions.

## Graphical Abstract

**Graphical Abstract:**
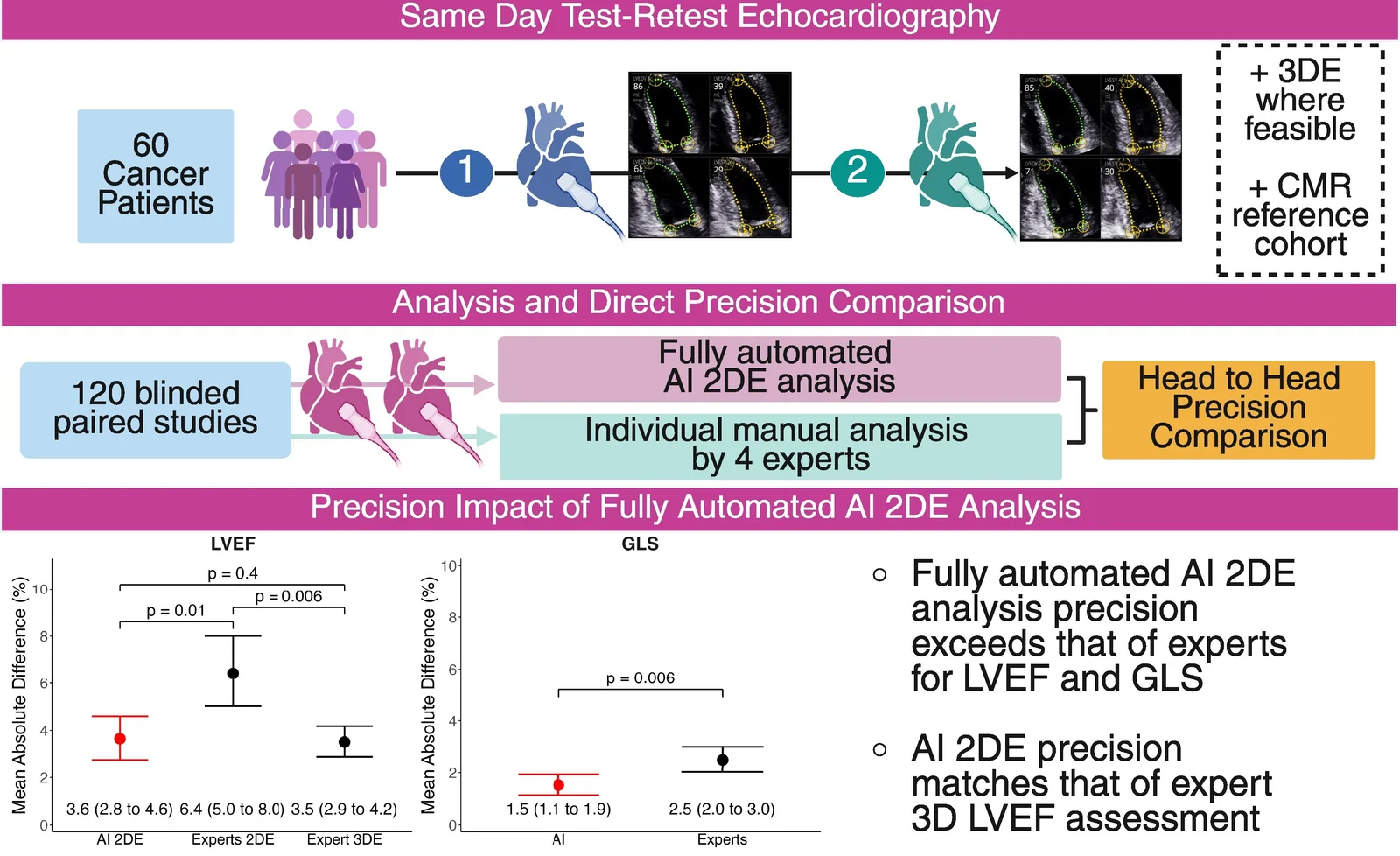
Fully automated analysis of cardiotoxicity surveillance echocardiograms: A direct test–retest precision comparison with experts. A prospective observational clinical trial of transthoracic echocardiography measurement precision in patients with cancer. Fully automated AI analysis improves precision compared with manual expert analysis; 2DE = 2D echocardiography, 3DE = 3D echocardiography, AI = artificial intelligence, GLS = global longitudinal strain, LVEF = left ventricular ejection fraction, MAD = mean absolute difference.

## Introduction

Cardiovascular surveillance is mandated in cancer patients receiving cardiotoxic cancer therapies to enable early detection and management of cancer therapy-related cardiac dysfunction (CTRCD).^1,2^ Serial cardiac imaging plays a critical role, with transthoracic echocardiography (TTE) recommended as the first-line modality for measurement of left ventricular ejection fraction (LVEF) and global longitudinal strain (GLS).^1,3^ 3D echocardiographic (3DE) assessment of LVEF is advised, although feasibility in cardio-oncology cohorts has been reported as only moderate.^4,5^ Symptomatic and severe asymptomatic CTRCD is uncommon with modern cardiotoxic therapy regimes,^6^ with most events detected via asymptomatic changes in LVEF and/or relative decline in GLS across repeated studies. Clinicians must be confident that measured differences represent true CTRCD, rather than measurement variability, as cardiac imaging results gatekeep major clinical decision-making, including cancer treatment continuation vs. interruption. The clinically necessary test performance to achieve this requires the detection of changes that are relatively small (an absolute change in LVEF ≥ 10% and a relative change in GLS ≥ 15%) compared with current measurement precision. Therefore, reducing sources of noise and improving measurement precision is critical to care. Previous studies of echocardiographic precision in cardio-oncology populations have calculated the minimal detectable change (MDC) (the lowest value where, with 95% certainty, a true change is being observed) for LVEF by 2D echocardiography (2DE) to be 9.9–13% when, as commonly occurs in clinical practice, sequential surveillance scans are reported by two different expert observers.^7,8^ Similarly, the absolute MDC for GLS by 2DE has been reported as up to 2.7%.^7,8^

The use of artificial intelligence (AI) analysis in cardiovascular magnetic resonance (CMR) may deliver superior measurement precision, for assessing cardiac volumes and function, compared with expert human readers;^9,10^ however, provision is currently limited. Fully automated AI analysis of echocardiography has demonstrated parity in precision with expert human readers for linear measurements of the LV wall thickness^11^ and superior precision for estimation of LV mass,^12^ but its wider impact on functional measurement precision has not been assessed.

We directly compared the precision of fully automated AI and expert manual analysis of 2DE in a clinical cohort of at-risk cancer patients, utilizing a same-day ‘test–retest’ study design to minimize sources of variation in echocardiographic results and maximize the precision impact of analysis method specifically.

## Methods

### Study design

**Figure 1:**
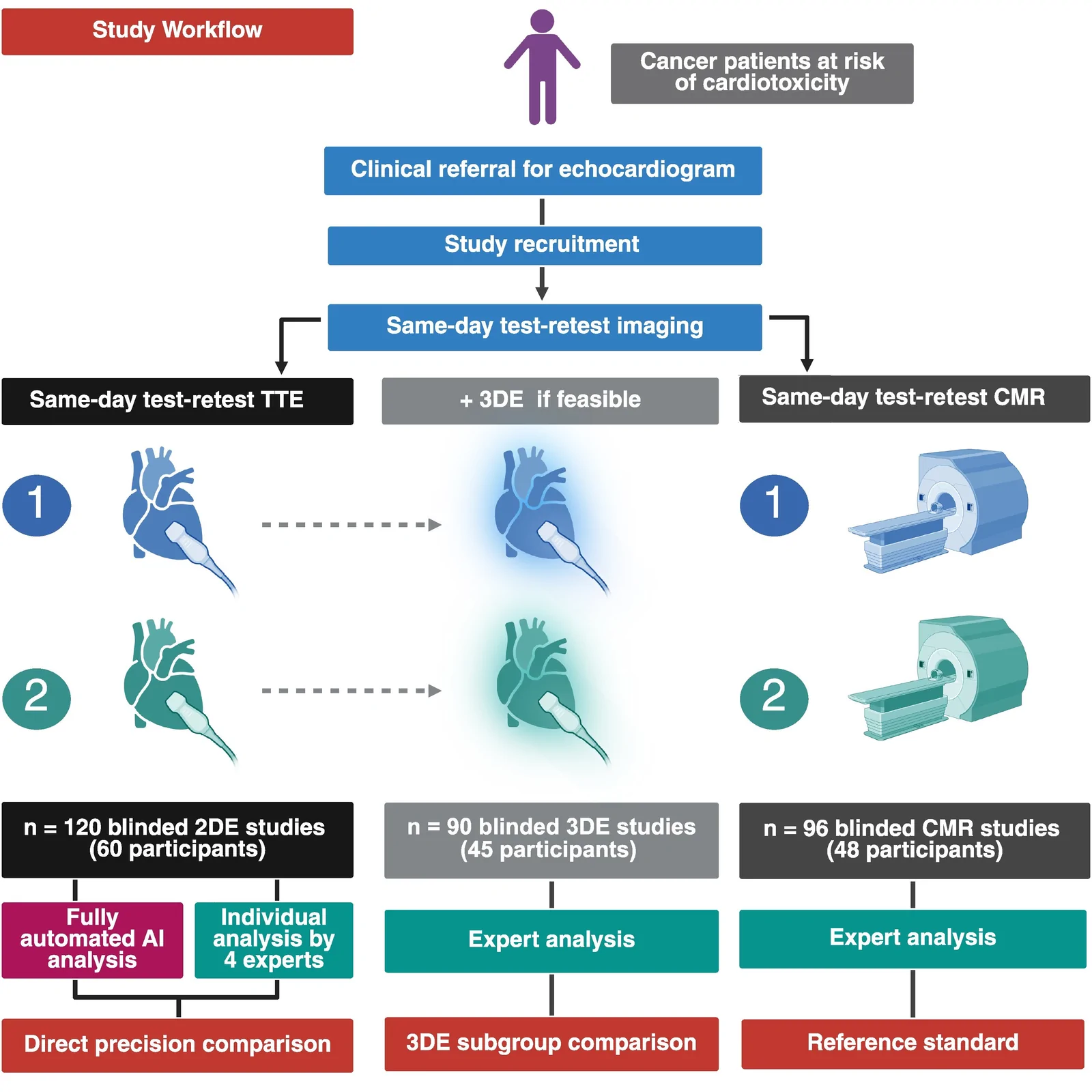
Study workflow. Clinical oncology patients underwent same-day repeat TTE prior to blinded analysis by experts and fully automated AI; subgroups also underwent paired 3DE and CMR on the same day. 3DE = 3D echocardiography, AI = artificial intelligence, CMR = cardiovascular magnetic resonance, TTE = transthoracic echocardiography.

A prospective observational clinical trial of measurement precision (NCT06817044). Consecutive patients referred for cardiotoxicity surveillance at Barts Heart Centre, London, UK, were prospectively recruited. Participants, who had been referred for TTE as part of usual clinical care, underwent repeat same-day (‘test–retest’) studies (*Figure 1*; Supplementary data online, *Figure S1*). This approach facilitates the most direct comparison of 2DE analysis method precision, by reducing confounding biases observed with repeat testing on different days. Inclusion criteria were age > 18 years old and a pre-existing clinical request for a transthoracic echocardiogram as part of cardiotoxicity surveillance. Exclusion criteria were atrial fibrillation or a high burden of ventricular ectopy.

### Ethical approvals

The study was approved by the UK National Research Ethics Service (16/LO/1815) and registered with ClinicalTrials.gov (NCT06817044) and was conducted in accordance with the principles of the Declaration of Helsinki. All participants gave written informed consent.

### Echocardiography protocol

2DE and 3DE were performed (Vivid E9, GE, Horten, Norway) according to the American Society of Echocardiography protocols.^13^ Image settings were optimized with harmonics on and a frame rate of 80 frames per second for 2DE. A 3D full volume dataset of the ventricle was obtained with gated (4 beats) acquisition with sector size, depth, and number of heartbeats optimized to obtain the highest possible volume rates (>40 frames per second).

All repeat studies were completed within 1 h to avoid physiological changes in loading conditions, with patients not permitted to eat or drink between studies. Repeats were performed as a new study by the same operator utilizing the same equipment. Between studies, participants were mobilized and repositioned as per usual practice. No measurements were performed during acquisition. Images were saved digitally in DICOM format following acquisition. All ‘test–retest’ pairs were separated and anonymized prior to analysis.

### Expert manual analysis

Four human experts served as the 2DE standard (accreditations and training; Supplementary data online, *Supplemental Methods*). Readers were blind to participant ID and study sequence. Manual analysis was performed using a validated web-based reporting platform,^11,14^ without automated assistance. Each expert was presented and encouraged to report every study, unless image quality made it unfeasible. 2D left ventricular (LV) volumes and LVEF were calculated using Simpson’s biplane method. Strain was calculated as the change in endocardial curve length (measured from the septal mitral annular hinge point to the lateral mitral annular hinge point via the endocardial apex) between end-diastole and end-systole, expressed as a percentage of the end-diastolic curve length for the apical four-, three-, and two-chamber views and GLS defined as the mean of the 3.^14,15^ 3D volumetric analysis was performed by a single expert reader with basal and apical guide points placed in the end-diastolic and end-systolic frames (EchoPAC version 113, GE).

### Fully automated AI analysis

Images underwent unsupervised automated AI analysis (EchoConfidence, MyCardiumAI).^14^ Following unsupervised view classification all available heart beats were analysed, with automated exclusion of low confidence measurements, providing an averaged output for each metric. LV volumes and LVEF were calculated using Simpson’s modified biplane method with myocardial strain automatically measured using endocardial curve length.^14,15^ No manual correction occurred.

### Image quality assessment

All studies were scored as ‘good’ or ‘suboptimal’ by a separate independent expert, based on published criteria (see Supplementary data online, *Supplemental Methods*).^16^

### Reference standard—CMR

CMR offers gold-standard precision and accuracy.^7-9^ Therefore, as a comparative reference standard and to properly contextualize the impact of AI analysis on echocardiographic precision, a subgroup of participants underwent test–retest CMR on the same day as their test–retest echocardiography (1.5 T Magnetom Aera, Siemens AG Healthcare, Germany). CMR cine images (three long-axis views and a short-axis stack) were acquired using a retrospectively electrocardiogram (ECG)-gated balanced steady-state free-precession sequence during end-expiratory breath-holding. Blinded analysis was performed using CVI42 software (Circle Cardiovascular Imaging, Alberta, Canada) by an expert reader. Contiguous SAX slices were segmented using a semi-automated thresholding technique to derive LV volumes and LVEF.

### Primary outcomes

The primary outcome measure was mean absolute difference (MAD) in 2DE-derived metrics of LV function (LVEF and GLS) between test–retest scans.

Assuming a two-sided *α* of 0.05 and 60 paired observations, the study had 90% power to detect a difference in test–retest MAD of 1.9 EF units and 0.9 GLS units between analysis methods.

### Secondary outcomes

Exploratory analysis was performed in groups stratified by independent 2DE quality assessment (good or suboptimal). Comparative analyses of precision and accuracy were conducted in participants with feasible paired 3DE and CMR imaging; there was no AI analysis of 3DE or CMR.

### Statistical analyses

Statistical analyses were performed in R (v2025.09.1, R Studio, Boston, MA) using the dplyr, tidyr, purrr, and boot packages. Continuous variables are expressed as mean ± SD. Expert results are presented as the average across four readers unless otherwise stated. Precision metrics and confidence intervals (CIs) were estimated using non-parametric bootstrap resampling (10 000 iterations, fixed seed).

Precision was assessed by (i) MAD between same-day repeat scan pairs, with significance testing by Wilcoxon signed-rank test; (ii) MDC, defined as twice the standard error of measurement (SEM), where SEM was estimated as the root mean squared error of a one-way analysis of variance (ANOVA) model^7,8^; (iii) within-subject coefficient of variation (WSCV), calculated by the root mean squared approach^17^; (iv) Bland–Altman analysis; (v) ordinary least squares regression (AI = *β*_0_ + *β*_1_·expert), with proportional bias assessed by testing *β*_1_ = 1; and (vi) Pearson correlation coefficients with 95% CIs derived by Fisher’s z-transformation. Further details are provided in the Supplementary data online, *Supplemental Methods*.

Agreement with the CMR reference standard was assessed using MAD, WSCV, Bland–Altman analysis, and correlation coefficients. Intraclass correlation coefficients were not used given known systematic differences in absolute values between CMR and TTE.^18^

Sample size estimates were derived from the standardized difference (*d*) in each metric, assuming 90% power and *α* = 0.05, where *d* is the target clinical change divided by the SD of test–retest differences.

Anonymized data and analytic materials are available upon reasonable request.

## Results

Sixty patients were recruited; their clinical characteristics are summarized in *Table 1* and echocardiographic characteristics in *Table 2*. Three (5%) participants had prior clinically significant CTRCD, with a mean LVEF of 41% (±3) and a mean GLS of −13.0% (±3.1) at assessment.

**Table 1:** Characteristics of study participants

	n = 60
Age (mean ± SD) (years)	52 ± 12
Female	50 (83)
Body mass index (BMI) (mean ± SD) (kg/m^2^)	27.7 ± 6.2
BMI > 25 to <30	22 (37)
Obese (BMI ≥ 30)	16 (27)
Body surface area (BSA) (mean ± SD) (m^2^)	1.81 ± 0.2
Sinus rhythm	60 (100)
Primary tumour	
Breast cancer	37 (62)
Lymphoma	8 (13)
Myeloma	6 (10)
Other haematological malignancy	4 (7)
Gastro-intestinal malignancy	2 (3)
Other cancer	3 (5)
Prior mastectomy	17 (28)
Prior radiotherapy	18 (30)
Anthracycline treatment	38 (63)
Anti-HER2 treatment	29 (48)
Assessment stage	
Pre-cancer treatment	7 (12)
Peri-treatment	23 (38)
Post-treatment	30 (50)
Cardiovascular comorbidities	
Hypertension	18 (30)
Diabetes mellitus	1 (2)
Smoker (current or former)	9 (15)
Dyslipidaemia	6 (10)
Pre-existing cardiovascular disease	2 (3)
HFA-ICOS CV risk classification	
Low	36 (60)
Moderate	16 (27)
High/very high	8 (13)
Self-reported ethnicity	
Asian	13 (22)
Black	11 (18)
White	32 (53)
Other	4 (7)

Values presented as *n* (%) unless otherwise stated.

BMI = body mass index, BSA = body surface area, CV = cardiovascular, HFA-ICOS = Heart Failure Association—International Cardio-Oncology.

**Table 2:** Echocardiographic characteristics

Metric	AI analysis	Expert analysis	Correlation (*n* = 58 pairs)	*P* (correlation)
EDV (mL)	95 (± 25)	96 (± 26)	0.91 (0.84–0.84)	1.9 × 10^−21^
ESV (mL)	38 (± 15)	41 (± 17)	0.86 (0.77–0.92)	2.8 × 10^−17^
LVEF (%)	60 (± 7)	57 (± 7)	0.74 (0.60–0.84)	8.1 × 10^−11^
GLS (%)	−17.7 (±2.9)	−17.9 (±3.3)	0.69 (0.57–0.78)	6.1 × 10^−15^

Echocardiographic characteristics stratified by analysis method: values presented as mean ± SD or *n* (%). Pearson correlation coefficients are reported with two-sided *P* values and 95% CIs derived using Fisher’s z-transformation, together with the number of paired observations.

AI = artificial intelligence, EDV = end-diastolic volume, ESV = end-systolic volume, GLS = global longitudinal strain, LVEF = left ventricular ejection fraction.

One hundred and twenty paired scans underwent blinded analysis. Following independent review, 12 (20%) participants were deemed to have at least one scan with suboptimal image quality for LVEF assessment by Simpson’s biplane and 16 (26%) suboptimal image quality for GLS assessment. Experts were unable to measure LVEF in four of 120 individual scans (3%) and GLS in nine (8%). AI analysis failed to provide an LVEF measurement in four scans (3%) and GLS in four studies (3%). All failures occurred in studies identified as ‘suboptimal’ image quality. Average expert analysis was 6 ± 2 min per study; pooled AI analysis of 120 studies was completed in <2 min.

There was a good correlation between AI and expert 2DE measurements (LVEF *r* = 0.74, GLS *r* = 0.76), with a bias such that AI LVEF readings were higher than expert [mean 61% (± 7) vs. 57% (± 7), *P* = 0.008] (*Table 2*). Regression analysis demonstrated systematic and proportional differences between AI-derived and expert-derived measurements, as well as between expert readers **(**see Supplementary data online, *Table S1* and *Figure S2*). Comparison with expert analysis of CMR (*n* = 48, acquired on the same day as the test–retest TTE) was performed as a reference standard; AI 2D analysis demonstrated significantly higher agreement with CMR than expert 2D analysis [LVEF MAD 5.0% (CI 4.1–5.9) vs. 7.8% (CI 6.6–9.0) *P* = 0.006; LVEF *r* = 0.70 (CI 0.57–0.79) vs. 0.56 (0.48–0.62), *P* = 0.01] (see Supplementary data online, *Table S2* and *Figure S3*).

**Figure 2:**
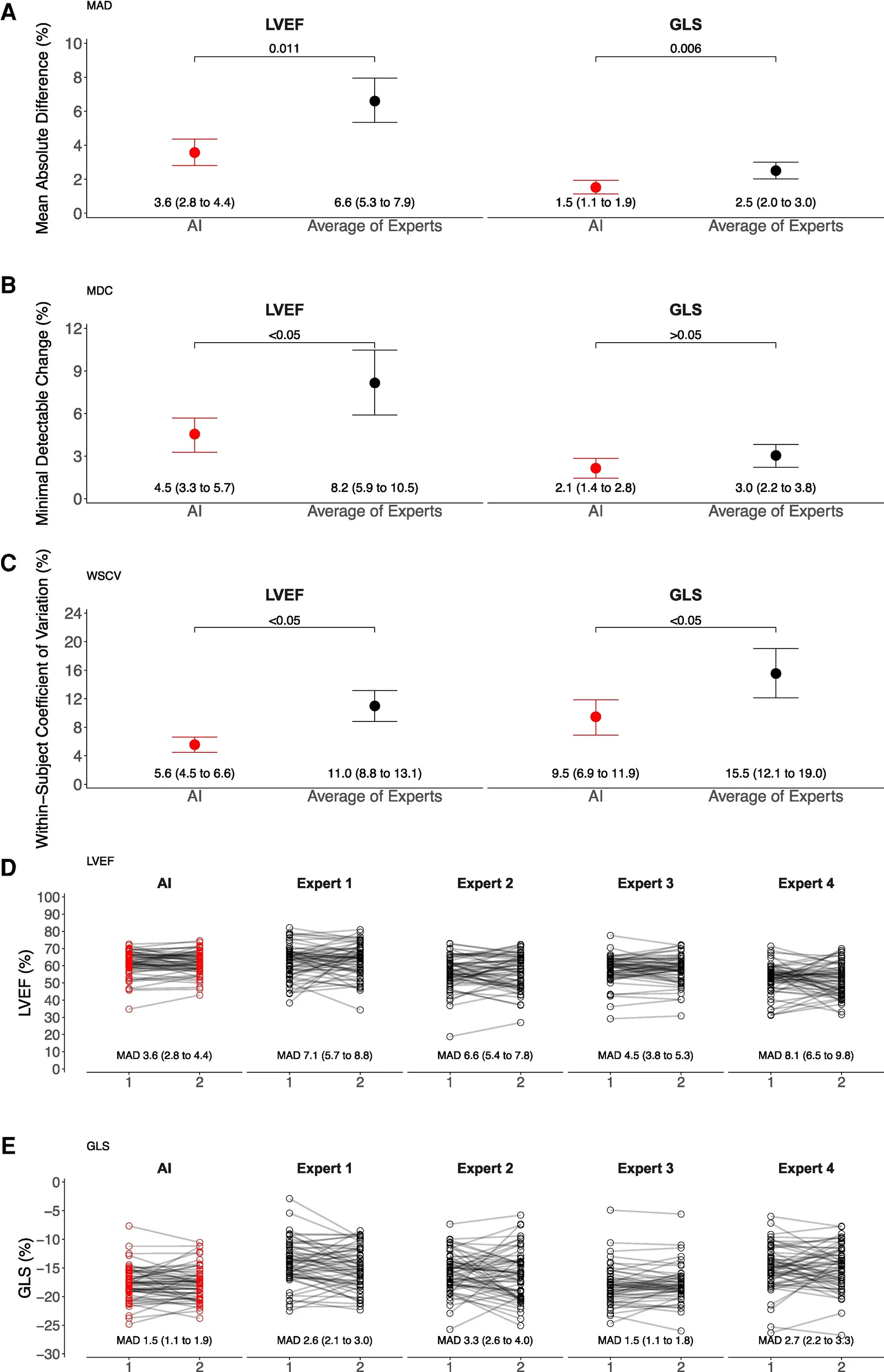
Precision comparison of AI vs. expert analysis of 2DE test–retest studies. (*A*) LVEF and GLS MAD, with 95% CIs, for test–retest studies. (*B*) LVEF and GLS MDC with 95% CI. (*C*) LVEF and GLS within subject coefficient of variation with 95% CI. (*D*) Individual reader LVEF results across test–retest studies, with individual MAD and 95% CI. (*E*) Individual reader GLS results across test–retest studies, with individual MAD and 95% CI. AI = artificial intelligence, GLS = global longitudinal strain, LVEF = left ventricular ejection fraction, MAD = mean absolute difference, MDC = minimal detectable change, WSCV = within-subject coefficient of variation.

**Figure 3:**
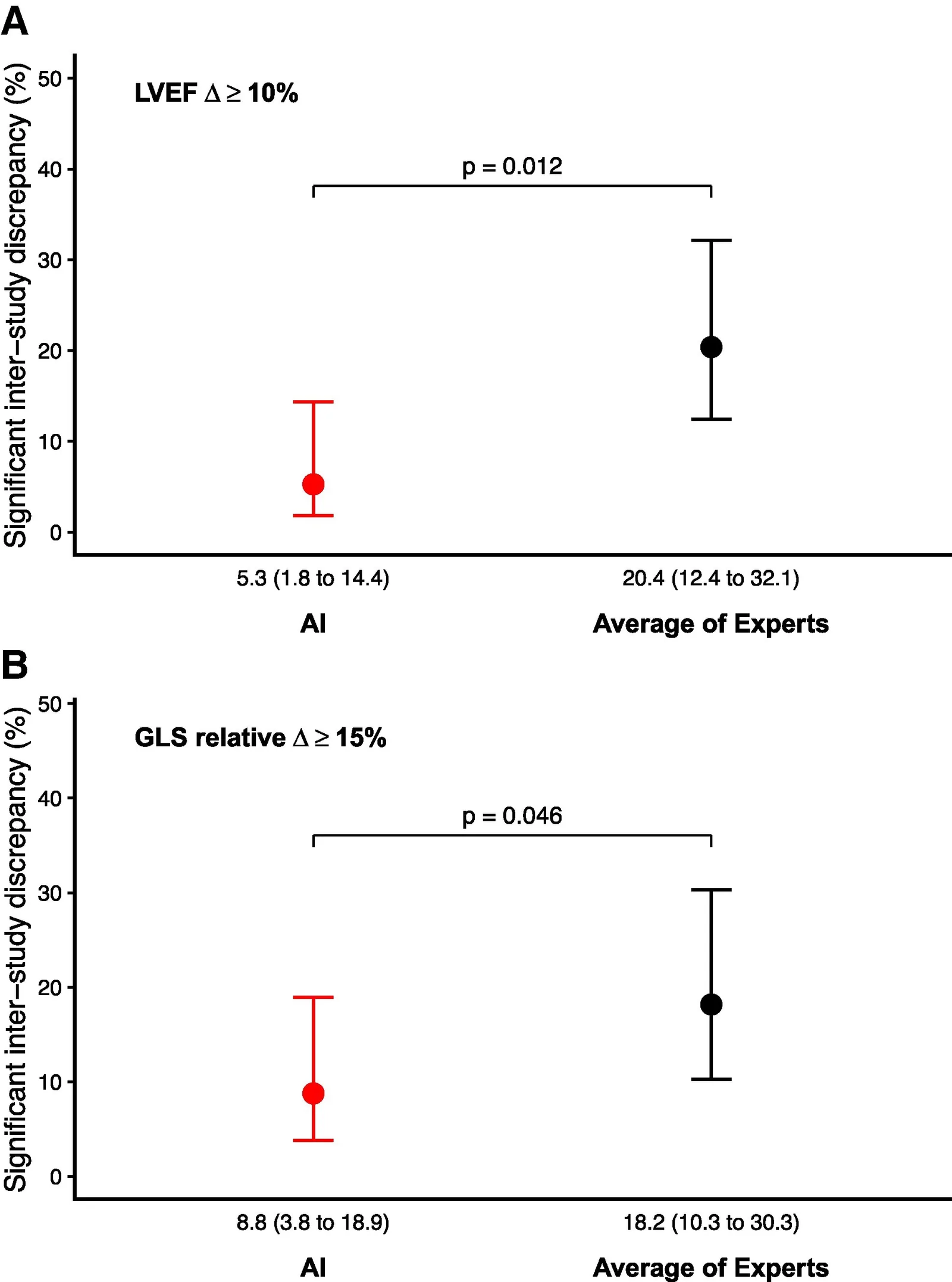
Significant inter-study discrepancy for AI vs. expert analysis of 2DE test–retest studies. (*A*) The percentage of 2D echocardiography test–retest pairs with LVEF discrepancy ≥ 10% for AI and expert analysis, with CIs. (*B*) The percentage of 2D test–retest pairs with relative GLS discrepancy ≥ 15% for AI and expert analysis, with CIs. AI = artificial intelligence, GLS = global longitudinal strain, LVEF = left ventricular ejection fraction.

We compared precision between fully automated AI and expert analysis of test-retest 2DE studies (*Figure 2*; Supplementary data online, *Tables S3*–*S5* and *Figure S4*). AI 2DE analysis demonstrated significantly lower MAD than experts for both LVEF [3.6% (2.8–4.4) vs. 6.6% (5.3–7.9), *P* = 0.011] and GLS [1.5% (1.1–1.9) vs. 2.5% (2.0–3.0), *P* = 0.006], with numerically lower values than all four experts individually. CMR-derived LVEF MAD was significantly lower than expert and AI 2DE LVEF MAD [2.0% (1.7–2.4), *P* = 0.0023 and 0.013, respectively (Supplementary data online, *Table S6*)]. MDC was lower for AI than experts [LVEF 4.5% (3.3–5.7) vs. 8.2% (5.9–10.5), *P* < 0.05]. The incidence of significant inter-study discrepancy (LVEF absolute change ≥ 10% or GLS relative change ≥ 15% between test–retest pairs) was significantly lower for AI than expert analysis; LVEF 5.1% (1.7–13.9) vs. 20.6 (12.6–32.2), *P* = 0.012; GLS 8.5% (3.7–18.3) vs. 18.4 (10.6–30.4), *P* = 0.046 (*Figure 3*; Supplementary data online, *Table S7* and *Figures S5* and *S6*).

Subgroup analysis of precision by image quality (‘good’ and ‘suboptimal’) was performed (*Table 3*; Supplementary data online, *Tables S4* and *S5*). In good-quality studies (*n* = 48), 2DE AI MAD remained significantly lower than 2DE experts for LVEF [3.4% (2.7–4.1) vs. 6.4% (5.0–7.8), *P* = 0.027] and GLS [1.2% (0.9–1.7) vs. 2.4% (1.9–3.0), *P* = 0.021]. Differences were numerically preserved but non-significant in suboptimal studies (*n* = 12). The decline in precision related to suboptimal image quality was numerically greater for expert than AI analysis [expert LVEF MAD +1.6 (0–3.2) vs. AI + 0.6 (−0.4–1.7), *P* = 0.34; Supplementary data online, *Figure S7*]. There was no significant difference in AI vs. expert measurement precision based on the baseline Heart Failure Association—International Cardio-Oncology cardiovascular risk classification or other participant characteristics (see Supplementary data online, *Table S8* and *Figure S7*).

**Table 3:** Precision stratified by scan quality

Mean absolute difference	AI analysis	Expert analysis	*P*
Good quality			
LVEF (%)	3.4 (2.7–4.1)	6.3 (5.0–7.8)	0.027^a^
GLS (%)	1.2 (0.9–1.7)	2.4 (1.9–3.0)	0.021^a^
Suboptimal quality			
LVEF (%)	4.0 (2.3–5.8)	7.9 (5.0–11.0)	0.09
GLS (%)	1.2 (0.6–2.1)	2.6 (1.7–3.7)	0.2

Precision stratified by analysis method and scan quality—assessed by a blinded, independent expert observer.

AI = artificial intelligence, EDV = end-diastolic volume, ESV = end-systolic volume, GLS = global longitudinal strain, LVEF = left ventricular ejection fraction.

^a^Denotes statistical significance (*P* < 0.05).

**Figure 4:**
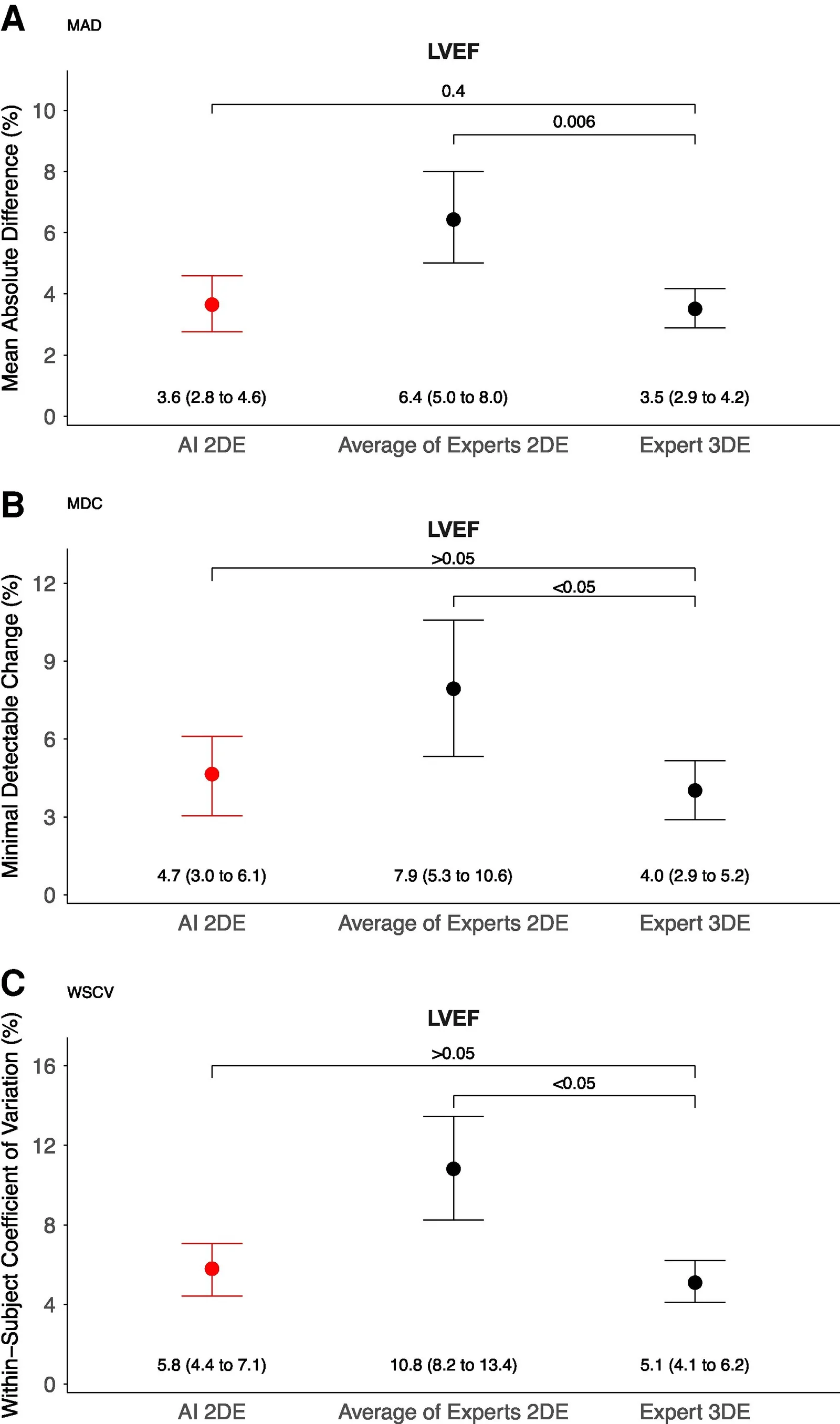
Precision comparison of AI 2DE vs. expert 2DE and 3DE analysis of test–retest studies. (*A*) LVEF MAD, with 95% CIs, for test–retest studies stratified by analysis method (*n* = 45 subgroup with 3DE). (*B*) LVEF MDC with 95% CI. (*C*) LVEF within subject coefficient of variation with 95% CI. 2DE = 2D echocardiography, 3DE = 3D echocardiography, AI = artificial intelligence, LVEF = left ventricular ejection fraction, MAD = mean absolute difference, MDC = minimal detectable change, WSCV = within-subject coefficient of variation.

Expert 3DE analysis demonstrated significantly improved LVEF precision compared with expert 2DE analysis [3.6% (2.8–4.6) vs. 6.4% (5.0–8.0), *P* = 0.006], but no significant difference to 2D AI LVEF analysis [3.5% (2.9–4.2), *P* = 0.4] (*Figure 4*; Supplementary data online, *Table S9*). The MDC for 3DE-derived LVEF [4.0 (CI 2.9–5.2)] was not significantly different than the MDC from AI analysis of 2DE [4.7% (3.0–46.1), *P* > 0.05] and was significantly lower than expert analysis of 2DE [7.9% (5.3 −10.6), *P* < 0.05].

## Discussion

In this first study of the impact of fully automated AI analysis on measurement precision for cardiotoxicity surveillance, we show improved precision compared with experts for quantification of LVEF and GLS. AI measurement of LVEF and GLS was feasible in 96% of patients, including 75% of those deemed to have inadequate quality images by an independent expert human reader, and compared with overall 92% feasibility by expert human analysis. For patients where 3DE datasets were available, the precision of AI-analysed 2D LVEF was not significantly different to the precision of 3DE LVEF, both of which were significantly better than expert manual 2D LVEF analysis. AI 2DE analysis demonstrated stronger agreement with the CMR reference standard than expert 2DE analysis.

### Measurement precision in cardiotoxicity surveillance

Cardiotoxicity surveillance for cancer patients receiving potentially cardiotoxic cancer therapies relies on the detection of small relative changes in markers of cardiac function over time. Confidently identifying such relative changes is essential for early detection and treatment of CTRCD and to avoid unnecessary interruptions to life-saving cancer therapies. This is difficult as the relative diagnostic changes border the precision of current measurement approaches; ultimately, contemporary expert analysis has a low signal-to-noise ratio that confounds our ability to detect meaningful change (see Supplementary data online, *Tables S10* and *S11*). Improving monitoring for cardiotoxicity, therefore, demands imaging biomarkers and analysis techniques that provide optimal consistency and feasibility.

There are limited large-scale published data regarding measurement precision in cardiotoxicity surveillance. Observed measures of precision also vary and may not be directly comparable. Lambert *et al.*^7^ reported cardiac imaging precision in a cohort of 30 healthy volunteers and 30 cancer patients. Participants underwent three serial TTE at 3-month intervals, with inter-observer test–retest 2DE LVEF MDC (two reporters analysing sequential studies) reported as 9.9%. Our expert manual analysis results are similar; 2DE LVEF MDC between expert readers (inter-observer precision) was 6.7–9.8% (see Supplementary data online, *Table S6*). There are currently no published data for same-day reproducibility testing for echocardiography in cancer patients. Across a mixed cohort (*n* = 24) of healthy volunteers and men with prior myocardial infarction, the MDC for test–retest 2D LVEF was 8.5%.^19^ Our cohort MDC for 2D LVEF by expert analysis was similar, 8.2%.

Current guidelines recommend assessment of LVEF with 3D echocardiography (3DE) where feasible;^1,2,20^ however, real-world clinical data have highlighted the challenges of implementation. O'Driscoll *et al.*^5^ assessed the feasibility of echocardiographic measurements across 105 consecutive referrals to a clinical cardio-oncology service. Quantitative LVEF assessment using Simpson’s biplane analysis by 2DE was feasible in 81%, with 3D LVEF measurement in only 40%. Similar results were found in another study, where feasibility of 3DE LVEF was 66% of breast cancer patients undergoing cardiotoxicity surveillance.^4^ Across our full cohort, human experts were able to report quantitative measurements for 2DE LVEF and GLS outputs in 92% of scans compared with 96% scans by AI analysis, with manual 3DE LVEF quantification feasible in 74% (see Supplementary data online, *Table S12*). In line with published evidence, we observed that expert LVEF measurement precision was higher with 3DE than 2DE (MDC 4.0% compared with 7.9%); however, AI analysis of 2DE LVEF was not significantly different to expert 3DE (4.7% compared with 4.0%). ‘One week’ test–retest scans have previously reported a MDC of 4.8% for 3DE LVEF in a cohort of 10 healthy volunteers.^21^ As anticipated, expert CMR LVEF measurement was more precise than all echocardiographic methods in our cohort (MDC 2.5%). Given the similar measurement precision but significantly higher feasibility of LVEF quantification using AI analysis of 2DE when compared with 3DE, AI-based imaging solutions offer the potential to broaden access to appropriate imaging for cardiotoxicity surveillance across all patients and institutions.

GLS measurement is recommended in cardiotoxicity surveillance but can be challenging.^3,5,22,23^ The absolute MDC for GLS was 3% by experts and 2.1% by AI, with a cohort average GLS of −17.7%. A 3% absolute difference in GLS, the expert manual MDC, would therefore represent more than the threshold for the diagnosis of mild asymptomatic CTRCD (15% relative change in GLS). Given that echocardiographic strain-guided cardio-protection may ameliorate cardiotoxicity,^23^ the need to assess relative GLS change with confidence is apparent but may not be feasible in clinical practice. These results highlight the importance of GLS measurement precision in cancer patients and may aid future decision-making with regard to thresholds for CTRCD and utilization of cardio-protection.^3^

Measurement precision has direct clinical impacts. The incidence of significant inter-study discrepancy (LVEF absolute change ≥ 10% or GLS relative change ≥ 15% between same-day test–retest pairs) in our cohort was significantly lower with AI analysis. The improved precision of AI analysis would therefore also have yielded a lower theoretical incidence of (false-positive) CTRCD compared with expert analysis (8.5% vs. 23%) (see Supplementary data online, *Figure S6*). In practice, these significant test–retest differences, more common with less precise analysis and likely more profound over longer surveillance intervals, might be perceived as true change and lead to inappropriate clinical decision-making.

Measurement precision also has important consequences within clinical research. Improved precision and resultant confidence in repeated measures of cardiac function may be leveraged to facilitate smaller sample sizes. Davies *et al.*^9^ described a potential 44% reduction in sample size, when AI compared with expert analysis was used to power a theoretical endpoint of >3% change in CMR-derived LVEF. Our findings replicate this; the improved precision of AI analysis could allow up to a 67% reduction in sample size compared with manual analysis, to appropriately power a theoretical endpoint of >5% 2DE-derived LVEF decline (see Supplementary data online, *Table S13*).

### AI in echocardiography

There are a growing number of commercially available AI software solutions for 2DE analysis. They have demonstrated utility in varied conditions, including heart failure, valvular heart disease, and amyloidosis.^24–27^ This is the first direct comparison of measurement precision between expert manual and unsupervised, fully automated AI analysis of LVEF and GLS. Salte *et al.*^28^ reported an improvement in GLS test–retest precision specifically when utilizing AI analysis in patients hospitalized with suspected acute coronary syndromes. Asch *et al.*^29^ reported lower inter-observer variability for 2DE-derived LVEF using supervised AI analysis. Sveric *et al.*^12^ recently reported increased accuracy and precision of 2DE-derived LV mass measurement utilizing fully automated analysis. Human-in-the-loop solutions with an ‘AI-assist’ approach may provide confidence for adopting AI 2DE analysis in routine clinical work, harnessing AI precision with the safety of expert human oversight.

### Limitations

This was a single-centre study, and a single AI software was employed. The study population had all been referred specifically for TTE. There was a low rate of pre-existing cardiac impairment or abnormality. Those with atrial fibrillation or a high burden of ventricular ectopy were excluded. The precision effects of ultrasound contrast agents, in cases of inadequate 2DE imaging, were not assessed and may have improved expert precision. We did not assess for other factors influencing inter-study reproducibility outside of the analysis method, though studies were acquired by the same operator using the same equipment within an hour, so the precision impact of acquisition factors was intentionally minimized. Longitudinal test–retest, rather than same-day, would reflect clinical cardiotoxicity surveillance, but this study was specifically designed for direct, focused comparison of 2DE analysis method precision, with maximal avoidance of other confounders. The direct impact on serial CTRCD detection cannot be assessed, though the strict precision comparison, isolating the impact of analysis method, indicates an improved signal-to-noise ratio with AI analysis that should facilitate the detection of true change. GLS was measured via endocardial border length shortening rather than speckle tracking, though this validated approach correlates well with speckle tracking and is utilized in multiple commercial AI packages.^14,15^ The exploratory analysis by suboptimal image quality had a small sample size and was not formally powered.

## Conclusion

Across cancer patients at risk of cardiotoxicity undergoing same-day test-retest TTE, fully automated AI analysis improved measurement precision and was feasible in almost all participants, even when image quality was poor. Practically, this translates to an ability to confidently detect a true LVEF change greater than 4.5% and a true GLS change greater than 2.1% when utilizing AI analysis, compared with 8.2% and 3.0%, respectively, by expert analysis. The use of AI analysis of 2DE aligns the measurement precision with 3DE whilst improving access due to higher feasibility. Leveraging this increased precision has the potential to provide wide benefits in cardiotoxicity surveillance and could help tackle long-standing health inequalities faced by many cancer patients who lack access to CMR or 3DE.

## Supplementary Material

qyag142_Supplementary_Data

## Data Availability

The data underlying this article will be shared on reasonable request to the corresponding author.

## Lead author biography

Dr Benjamin Dowsing, MRCP, MBBS, MSc, BSc (Hons), is a British Heart Foundation-funded research fellow and academic cardiology trainee at University College London and Barts Heart Centre, currently completing a PhD utilizing advanced cardiac imaging in cancer survivors.
