# Supplementary material for: Fully automated analysis of cardiotoxicity surveillance echocardiograms: a direct test–retest precision comparison with experts
Source: Eur Heart J Imaging Methods Pract. 2026 Aug 21;4(3):qyag142. doi: 10.1093/ehjimp/qyag142 (PMC13547863; doi:10.1093/ehjimp/qyag142)
Supplement: Supplementary file 1 [file qyag142_supplementary_data.docx]

**Fully automated analysis of cardiotoxicity surveillance echocardiograms:**

**A direct test-retest precision comparison with experts**

**Supplemental Appendix**

**Supplemental Methods 1:** [**Expert’s professional experience**]

All experts held professional certification in adult echocardiography through one or more recognized accrediting bodies, including the British Society of Echocardiography, Cardiovascular Credentialing International in cardiac echocardiography and vascular ultrasound, or the American Registry of Diagnostic Medical Sonography. Readers had extensive experience in the acquisition, interpretation, and reporting of adult transthoracic echocardiograms and remained actively engaged in routine clinical practice (median 7 years’ post accreditation specialist experience). All experts were also familiar with the reporting platform (formal training and >2 years’ experience).

**Supplemental Methods 2:** [**Image Quality Assessment]**

Individual studies were categorized as ‘suboptimal’ if they met the following criteria:

- 2DE: Poor endocardial definition in more than 2 segments and apical descent >1 cm (foreshortening).
- 3DE: More than two segments with poor endocardial tracking.
- GLS (2DE): Two or more segments with poor tracking in any of the 3 apical windows and 1cm of apical descent

**Supplemental Methods 3:** [**Statistical Analyses]**

1. Mean absolute difference (MAD):

$$MAD=\left( 1/n \right)\sum\left( i=1 \right)^{n}\left| x_{1i}-x_{2i} \right|$$

2. Minimal detectable change: The smallest change between two measurements that can be interpreted as real with 95% certainty. MDC was calculated as twice the standard error of measurement (SEM)^7,8^. SEM was estimated as the root mean squared error of a one-way analysis of variance model (MSE_within_)^7,8^.

$$MSE_{within}=\frac{1}{N-n}\sum_{i=1}^{n} \sum_{j=1}^{k_{i}} \left( y_{ij}-\bar{y_{i}} \right)^{2}$$

$$SEM=\sqrt{MSE_{within}}$$

3. Within-subject Coefficient of Variation (WSCV) - Calculated by the root mean squared approach^17^.

$$WSCV=100\text{\%}\times\sqrt{\frac{2}{N}\sum_{i=1}^{N} \left( \frac{x_{i}-y_{i}}{x_{i}+y_{i}} \right)^{2}}$$

4. Regression Analyses - Ordinary least squares model to compare AI and expert measurements (AI = β₀ + β₁·Expert). Coefficients are reported with standard errors, t statistics, and p values. Proportional bias was assessed by testing β₁ = 1 using a t statistic (β₁ − 1)/SE(β₁) with residual degrees of freedom.

**Supplemental Table 1:** [**AI:Expert Regression Analysis**. AI = β₀ + β₁·Expert. Coefficients are reported with standard errors, t statistics, and p values. Proportional bias assessed by testing β₁ = 1 using a t statistic (β₁ − 1)/SE(β₁) with residual degrees of freedom]

| **Reader** | **Term** | **Estimate** | **Standard Error** | **T-value** | **p-value** |
| --- | --- | --- | --- | --- | --- |
| **LVEF** | | | | | |
| E1 | Intercept | 34.3 | 3.6129 | 9.49 | 4.20×10⁻¹⁶ |
| E1 | Slope | 0.43 | 0.0569 | 7.52 | 1.36×10⁻¹¹ |
| E2 | Intercept | 33.8 | 2.8435 | 11.87 | 1.19×10⁻²¹ |
| E2 | Slope | 0.49 | 0.0498 | 9.78 | 9.04×10⁻¹⁷ |
| E3 | Intercept | 26.9 | 3.4998 | 7.70 | 7.38×10⁻¹² |
| E3 | Slope | 0.6 | 0.0600 | 9.97 | 6.02×10⁻¹⁷ |
| E4 | Intercept | 39.1 | 3.1961 | 12.23 | 1.73×10⁻²² |
| E4 | Slope | 0.43 | 0.0602 | 7.01 | 1.80×10⁻¹⁰ |
| Pooled | Intercept | 38.1 | 1.5815 | 24.08 | 3.33×10⁻⁸³ |
| Pooled | Slope | 0.4 | 0.0272 | 14.86 | 5.17×10⁻⁴¹ |
| **GLS** | | | | | |
| E1 | Intercept | -11.1 | 0.8992 | -12.30 | 1.65×10⁻²² |
| E1 | Slope | 0.46 | 0.0597 | 7.69 | 6.13×10⁻¹² |
| E2 | Intercept | -10.2 | 0.9288 | -10.98 | 1.42×10⁻¹⁹ |
| E2 | Slope | 0.47 | 0.0562 | 8.32 | 2.19×10⁻¹³ |
| E3 | Intercept | -6.51 | 1.2117 | -5.37 | 5.41×10⁻⁷ |
| E3 | Slope | 0.61 | 0.0664 | 9.25 | 6.09×10⁻¹⁵ |
| E4 | Intercept | -9.7 | 0.8442 | -11.54 | 1.46×10⁻²⁰ |
| E4 | Slope | 0.53 | 0.0547 | 9.74 | 1.85×10⁻¹⁶ |
| Pooled | Intercept | -10.6 | 0.4726 | -22.38 | 2.09×10⁻⁷⁴ |
| Pooled | Slope | 0.45 | 0.0290 | 15.49 | 2.04×10⁻⁴³ |

AI = Artificial Intelligence, E1 -4 = Expert 1 – 4, EDV = End diastolic volume, ESV = End systolic volume, GLS = Global longitudinal strain, LVEF = Left ventricular ejection fraction

**Supplemental Table 2:** [**Agreement with CMR reference standard**. Accuracy and association comparison to CMR reference standard by TTE analysis method]

|  | **CMR expert vs 2DE AI**  **(n = 96)** | **CMR expert vs 2DE Expert**  **(n = 96)** | **CMR expert vs 3DE Expert**  **(n = 72)** | **P (CMR-2DE expert vs CMR-2DE AI)** |
| --- | --- | --- | --- | --- |
| **Mean Absolute Difference (95% CI)** | | | | |
| **LVEF (%)** | 5.0 (4.1 to 5.9) | 7.8 (6.6 to 9.0) | 5.3 (4.3 to 6.3) | 0.006* |
| **EDV (ml)** | 34.9 (30.8 to 39.3) | 35.5 (28.2 to 37.0) | 40.6 (36.2 to 45.0) | 0.21 |
| **ESV (ml)** | 16.3 (13.5 to 19.5) | 14.8 (12.3 to 17.6) | 15.2 (12.4 to 18.2) | 0.17 |
| **Within-Subject Coefficient of Variation (95% CI)** | | | | |
| **LVEF (%)** | 8.1 (6.5 to 9.6) | 12.3 (10.4 to 14.2) | 8.5 (7.1 to 9.8) | <0.05 |
| **EDV (ml)** | 24.3 (21.9 to 26.6) | 23.8 (21.2 to 26.3) | 28.4 (25.8 to 30.9) | >0.05 |
| **ESV (ml)** | 28.9 (25.3 to 32.5) | 27 (23.5 to 30.4) | 26.4 (22.7 to 30.1) | >0.05 |
| **Bland-Altman Limits of Agreement (95% CI)** | | | | |
| **LVEF (%)** | -13.1 to 13.0 | -8.6 to 16.6 | -9.2 to 15.0 | >0.05 |
| **EDV (ml)** | -10.2 to 77.7 | -8.7 to 75.8 | 2.8 to 78.1 | >0.05 |
| **ESV (ml)** | -17.0 to 46.1 | -18.2 to 39.7 | -12.2 to 40.8 | >0.05 |
| **Bias (95% CI)** | | | | |
| **LVEF (%)** | -0.06 (-1.43 to 1.32) | -8.6 to 16.6 | 2.92 (1.52 to 4.35) | - |
| **EDV (ml)** | 33.78 (29.27 to 38.47) | -8.7 to 75.8 | 40.50 (36.10 to 45.02) | - |
| **ESV (ml)** | 14.51 (11.28 to 17.82) | -18.2 to 39.7 | 14.28 (11.22 to 17.54) | - |
| **Correlation** | | | | |
| **LVEF (%)** | 0.70 (0.57 to 0.79) | 0.56 (0.48 to 0.62) | 0.76 (0.65 to 0.84) | 0.01* |
| **EDV (ml)** | 0.81 (0.73 to 0.87) | 0.76 (0.72 to 0.80) | 0.84 (0.74 to 0.89) | 0.3 |
| **ESV (ml)** | 0.84 (0.77 to 0.89) | 0.79 (0.74 to 0.82) | 0.88 (0.82 to 0.93) | 0.4 |

2DE = 2D echocardiography, 3DE = 3D echocardiography, AI = Artificial Intelligence, CI = Confidence Intervals, EDV = End diastolic volume, ESV = End systolic volume, GLS = Global longitudinal strain, LVEF = Left ventricular ejection fraction. *Denotes statistical significance (p<0.05)]

|  | **AI Analysis** | **Expert Analysis** | **p** |
| --- | --- | --- | --- |
| **Mean Absolute Difference (95% CI)** | | | |
| LVEF (%) | 3.6 (2.8 to 4.4) | 6.6 (5.3 to 7.9) | 0.011* |
| GLS (%) | 1.5 (1.1 to 1.9) | 2.5 (2.0 to 3.0) | 0.006* |
| EDV (ml) | 7.2 (5.8 to 8.9) | 10.9 (8.7 to 13.4) | 0.053 |
| ESV (ml) | 3.7 (2.9 to 4.5) | 7.1 (5.6 to 8.7) | 0.004* |
| **Minimal Detectable Change (95% CI)** | | | |
| LVEF (%) | 4.5 (3.3 to 5.7) | 8.2 (5.9 to 10.5) | <0.05* |
| GLS (%) | 2.1 (1.4 to 2.8) | 3.0 (2.2 to 2.8) | >0.05 |
| EDV (ml) | 9.1 (6.2 to 12.0) | 13.7 (9.5 to 17.6) | >0.05 |
| ESV (ml) | 4.7 (3.2 to 6.2) | 9.0 (5.9 to 12.2) | >0.05 |

**Supplemental Table 3: [Comparative Precision Metrics.** Comparison of precision between analysis methods]

AI = Artificial Intelligence, CI = Confidence Intervals, EDV = End diastolic volume, ESV = End systolic volume, GLS = Global longitudinal strain, LVEF = Left ventricular ejection fraction. *Denotes statistical significance (p<0.05)]

**Supplemental Table 4:** [**Within Subject Co-Efficient of Variation**. Comparison of within subject co-efficient of variation between analysis methods]

|  | **AI Analysis** | **Expert Analysis** |
| --- | --- | --- |
| EDV (%) | 6.9 (5.4 to 8.5) | 11.1 (8.6 to 13.5) |
| ESV (%) | 9.7 (7.7 to 11.6) | 16.4 (12.9 to 20.1) |
| LVEF (%) | 5.6 (4.5 to 6.6) | 11.0 (8.8 to 13.1) |
| GLS (%) | 9.5 (6.9 to 11.9) | 15.5 (12.1 to 19.0) |
| **Good Quality Subgroup** | | |
| EDV (%) | 6.5 (4.9 to 8.1) | 11.2 (8.4 to 13.8) |
| ESV (%) | 9.1 (7.2 to 11.0) | 16.4 (12.3 to 20.5) |
| LVEF (%) | 5.2 (4.1 to 6.3) | 10.6 (8.2 to 13.2) |
| GLS (%) | 9.1 (6.0 to 11.1) | 14.6 (11.6 to 17.5) |
| **Suboptimal Quality Subgroup** | | |
| EDV (%) | 6.4 (3.9 to 8.9) | 10.3 (6.0 to 14.4) |
| ESV (%) | 9.4 (4.0 to 14.3) | 16.8 (10.1 to 22.9) |
| LVEF (%) | 5.8 (3.5 to 7.7) | 12.2 (7.4 to 16.4) |
| GLS (%) | 9.7 (6.5 to 11.2) | 16.7 (8.5 to 24.3) |

AI = Artificial Intelligence, EDV = End diastolic volume, ESV = End systolic volume, GLS = Global longitudinal strain, LVEF = Left ventricular ejection fraction

**Supplemental Table 5:** [**Bland-Altman Limits of Agreement.** Comparison of Bland-Altman limits of agreement between analysis methods]

|  | **AI Analysis** | **Expert Analysis** |
| --- | --- | --- |
| EDV (ml) | -20.5, 16.1 | -29.6, 25.7 |
| ESV (ml) | -10.1, 9.0 | -18.9, 17.8 |
| LVEF (%) | -8.7, 8.3 | -16.5, 16.5 |
| GLS (%) | -3.6, 3.6 | -5.9, 6.4 |
| **Good Quality Subgroup** | | |
| EDV (ml) | -19.6, 13.9 | -30.8, 24.9 |
| ESV (ml) | -9.6, 7.8 | -18.9, 16.8 |
| LVEF (%) | -8.3, 8.2 | -16.0, 16.0 |
| GLS (%) | -3.6, 3.8 | -5.9, 6.1 |
| **Suboptimal Quality Subgroup** | | |
| EDV (ml) | -13.6, 19.5 | -22.1, 27.7 |
| ESV (ml) | -6.0, 10.5 | -19.0, 22.5 |
| LVEF (%) | -10.9, 9.1 | -18.6, 19.3 |
| GLS (%) | -3.9, 3.0 | -5.6, 6.5 |

AI = Artificial Intelligence, EDV = End diastolic volume, ESV = End systolic volume, GLS = Global longitudinal strain, LVEF = Left ventricular ejection fraction.]

**Supplemental Table 6 [CMR Precision Reference Standard**. Comparison of precision between analysis methods in n=48 subgroup with paired CMR]

|  | **CMR**  **Expert Analysis** | **2DE**  **Expert Analysis** | **2DE**  **AI Analysis** | **3DE**  **Expert Analysis**  **(n = 36)** | **P (CMR vs 2DE expert /2DE AI/ 3DE Expert)** |
| --- | --- | --- | --- | --- | --- |
| **Mean Absolute Difference (95% CI)** | | | | | |
| LVEF (%) | 2.0 (1.7 to 2.4) | 6.5 (5.1–8.0) | 3.9 (3.0–4.9) | 3.3 (2.7–3.9) | 0.0023 / 0.013 / 0.017 |
| EDV (ml) | 4.1 (3.7 to 5.3) | 10.7 (8.2–13.4) | 6.8 (5.0–8.7) | 8.9 (6.7–11.3) | 0.0018 / 0.022 / 0.014 |
| ESV (ml) | 3.0 (2.5 to 3.5) | 6.8 (5.2–8.6) | 3.3 (2.3–4.4) | 5.0 (3.8–6.4) | 0.0066 / 0.86 / 0.052 |
| **Minimal Detectable Change (95% CI)** | | | | | |
| LVEF (%) | 2.5 (1.8 to 3.1) | 8.0 (5.4–10.5) | 4.9 (3.4–6.3) | 3.7 (2.6–4.6) | <0.05 / <0.05 / >0.05 |
| EDV (ml) | 4.8 (3.7 to 5.9) | 13.3 (8.7–17.7) | 8.7 (5.6–11.5) | 10.8 (7.1–14.4) | <0.05 / >0.05 / <0.05 |
| ESV (ml) | 3.6 (2.7 to 4.6) | 8.5 (5.5–11.8) | 4.7 (2.5–6.9) | 6.2 (4.0–8.4) | <0.05 / >0.05 / >0.05 |
| **Within Subject Co-Efficient of Variation (95% CI)** | | | | | |
| LVEF (%) | 3.0 (2.5 to 3.6) | 11.0 (8.0–13.0) | 6.0 (5.0–7.0) | 4.6 (3.8–5.3) | <0.05 / <0.05 / <0.05 |
| EDV (ml) | 2.9 (2.4 to 3.4) | 11.0 (8.0–13.0) | 6.7 (5.0–8.4) | 9.5 (7.0–12.0) | <0.05 / <0.05 / <0.05 |
| ESV (ml) | 5.3 (4.3 to 6.3) | 15.6 (12.1–18.9) | 9.9 (6.9–12.7) | 12.7 (9.6–15.7) | <0.05 / <0.05 / <0.05 |
| **Bland Altman Limits of Agreement (95% CI)** | | | | | |
| LVEF (%) | -4.7, 5.2 | −16.5 to 16.1 | −10.4 to 9.5 | −7.7 to 7.2 | - |
| EDV (ml) | -10.0, 9.3 | −27.7 to 26.1 | −19.8 to 15.7 | −20.5 to 23.7 | - |
| ESV (ml) | -7.7, 6.8 | −17.4 to 17.4 | −10.1 to 9.2 | −12.0 to 13.6 | - |

2DE = Two-dimensional echocardiography, 3DE = Three-dimensional echocardiography, AI = Artificial Intelligence CMR = Cardiovascular Magnetic Resonance, EDV = End diastolic volume, ESV = End systolic volume, LVEF = Left ventricular ejection fraction

**Supplemental Table 7: [Significant Interstudy Discrepancy.** Comparison of significant LVEF and GLS discrepancy between AI and expert analysis of 2DE (n=60).

| **Outcome** | **AI** | **Average of Experts** | **Expert range (1→4)** |
| --- | --- | --- | --- |
| **LVEF Δ ≥10%** | 5.3% (1.8–14.4) | 20.4% (12.4–32.1) | 7.3 / 23.3 / 23.3 / 28.3% |
| **GLS relative Δ ≥15%** | 8.8% (3.8–18.9) | 18.2% (10.3–30.3) | 15.0 / 20.0 / 12.7 / 23.3% |

**Supplemental Table 8: [Precision analysis stratified by HFA-ICOS CV baseline risk.** Comparison of precision metrics in 2DE cohort (n=60) stratified by HFA-ICOS CV baseline risk.

|  | **Low**  (n=36) | **Medium**  (n=16) | **High** / **Very High**  (n=7) |
| --- | --- | --- | --- |
| **Mean Absolute Difference** | | | |
| EDV (ml) | 10.7 (7.6 to 14.3) | 10.8 (6.8 to 15.4) | 11.3 (7.2 to 16.0) |
| ESV (ml) | 6.5 (4.7 to 8.7) | 7.8 (4.4 to 11.9) | 7.3 (4.9 to 10.1) |
| LVEF (%) | 6.1 (4.5 to 7.8) | 7.4 (4.7 to 10.2) | 6.8 (4.6 to 9.2) |
| GLS (%) | 2.6 (1.9 to 3.4) | 2.4 (1.5 to 3.3) | 2.5 (1.7 to 3.3) |
| **Minimal Detectable Change** | | | |
| EDV (ml) | 12.8 (6.9 to 17.9) | 11.8 (3.4 to 18.5) | 13.3 (5.8 to 20.6) |
| ESV (ml) | 7.8 (4.2 to 11.6) | 9.3 (2.7 to 15.5) | 8.4 (3.8 to 12.7) |
| LVEF (%) | 7.3 (4.3 to 10.0) | 7.7 (2.3 to 12.6) | 7.8 (3.3 to 11.6) |
| GLS (%) | 3.1 (1.7 to 4.4) | 2.4 (0.8 to 3.9) | 2.7 (1.3 to 4.1) |
| **Within Subject Co-efficient of Variation** | | | |
| EDV (%) | 10.7 (7.3 to 13.9) | 10.9 (6.4 to 15.3) | 11.4 (7.4 to 15.1) |
| ESV (%) | 15.4 (11.0 to 19.5) | 17.8 (9.9 to 24.7) | 15.9 (11.2 to 20.2) |
| LVEF (%) | 9.3 (6.9 to 11.6) | 12.1 (7.6 to 16.1) | 12.0 (7.8 to 15.7) |
| GLS (%) | 15.1 (10.5 to 19.5) | 15.6 (8.8 to 22.0) | 15.4 (10.7 to 19.6) |
| **Bland-Altman Limits of Agreement** | | | |
| EDV (ml) | -30.9, 22.3 | -25.6, 27.3 | -29.4, 28.6 |
| ESV (ml) | -17.8, 14.9 | -21.7, 20.1 | -17.1, 19.2 |
| LVEF (%) | -15.1, 15.3 | -15.7, 19.2 | -18.2, 15.3 |
| GLS (%) | -6.6, 6.5 | -5.2, 5.7 | -5.1, 6.7 |

CV = Cardiovascular, EDV = End diastolic volume, ESV = End systolic volume, HFA-ICOS = Heart Failure Association – International Cardio-Oncology, GLS = Global longitudinal strain, LVEF = Left ventricular ejection fraction

**Supplemental Table 9: [3DE Cohort Extended Precision Metrics.** Comparison of precision metrics between analysis methods in (n = 45) cohort with 3D echocardiography]

|  | **AI 2DE Analysis** | **Expert 2DE Analysis** | **Expert 3DE Analysis** |
| --- | --- | --- | --- |
| **Mean Absolute Difference** | | | |
| EDV (ml) | 7.8 (6.0 to 9.8) | 10.7 (8.1 to 13.5) | 8.7 (6.9 to 10.7) |
| ESV (ml) | 3.7 (2.8 to 4.6) | 6.8 (5.2 to 8.6) | 5.0 (3.7 to 6.4) |
| **Minimal Detectable Change** | | | |
| EDV (ml) | 9.1 (5.8 to 12.4) | 13.2 (8.6 to 17.8) | 10.7 (7.3 to 13.9) |
| ESV (ml) | 4.6 (2.9 to 6.1) | 8.5 (5.4 to 12.0) | 6.7 (4.1 to 9.1) |
| **Within Subject Co-Efficient of Variation** | | | |
| EDV (%) | 7.4 (5.5 to 9.2) | 10.6 (8.0 to 13.2) | 9.3 (7.2 to 11.4) |
| ESV (%) | 9.5 (7.2 to 11.7) | 15.9 (12.2 to 19.6) | 13.1 (10.2 to 15.9) |
| **Bland-Altman Limits of Agreement** | | | |
| EDV (ml) | -22.5, 14.8 | -29.9, 24.2 | -20.2, 22.8 |
| ESV (ml) | -10.5, 8.1 | -18.4, 16.6 | -13.3, 13.8 |
| LVEF (%) | -7.8, 7.5 | -16.3, 16.1 | -8.0, 8.3 |

3DE= 3-dimensional echocardiography, AI = Artificial Intelligence, EDV = End diastolic volume, ESV = End systolic volume, GLS = Global longitudinal strain, LVEF = Left ventricular ejection fraction

**Supplemental Table 10: [Expert Inter-operator Precision.** Comparison of expert inter-operator precision]

|  | **E1/E2** | **E1/E3** | **E1/E4** | **E2/E3** | **E2/E4** | **E3/E4** |
| --- | --- | --- | --- | --- | --- | --- |
| **Mean Absolute Difference (95% CI)** | | | | | | |
| EDV (ml) | 17.7 (13.9 to 22.0) | 16.6 (13.5 to 19.9) | 33.6 (30.1 to 37.2) | 15.4 (12.6 to 18.3) | 20.6 (16.8 to 24.3) | 21.1 (17.8 to 24.8) |
| ESV (ml) | 9.6 (7.3 to 12.2) | 9.1 (7.4 to 11.0) | 8.8 (6.7 to 10.8) | 7.7 (6.0 to 9.7) | 7.6 (5.7 to 9.8) | 8.0 (6.4 to 9.9) |
| LVEF (%) | 8.4 (7.0 to 10.1) | 8.9 (7.2 to 10.6) | 12.1 (10.4 to 13.8) | 5.8 (4.9 to 6.7) | 7.2 (5.8 to 8.8) | 7.4 (5.8 to 9.2) |
| GLS (%) | 3.5 (2.8 to 4.3) | 4.4 (3.6 to 5.2) | 2.9 (2.3 to 3.6) | 3.3 (2.5 to 4.0) | 2.6 (2.1 to 3.2) | 3.8 (3.2 to 4.5) |
| **Minimal Detectable Change (95% CI)** | | | | | | |
| EDV (ml) | 18.5 (12.0 to 25.9) | 15.8 (11.1 to 21.1) | 13.7 (9.6 to 17.3) | 18.4 (13.3 to 22.7) | 16.8 (12.9 to 20.9) | 13.8 (9.2 to 18.8) |
| ESV (ml) | 13.1 (8.1 to 18.3) | 11.0 (7.8 to 14.2) | 10.7 (7.5 to 13.5) | 10.1 (6.2 to 13.8) | 9.6 (5.8 to 15.3) | 9.0 (6.3 to 11.6) |
| LVEF (%) | 8.5 (5.5 to 11.2) | 9.0 (6.2 to 11.6) | 9.8 (6.9 to 12.2) | 6.7 (5.1 to 8.1) | 8.3 (5.5 to 10.9) | 7.9 (5.2 to 11.3) |
| GLS (%) | 4.2 (2.9 to 5.5) | 3.3 (2.2 to 4.4) | 3.8 (2.6 to 4.8) | 3.4 (2.4 to 4.4) | 3.2 (2.2 to 4.1) | 3.3 (2.3 to 4.0) |
| **Within Subject Co-Efficient of Variation (95% CI)** | | | | | | |
| EDV (%) | 16.7 (13.3 to 19.8) | 14.1 (11.7 to 16.2) | 26.9 (24.8 to 28.8) | 14.5 (12.1 to 16.7) | 19.3 (16.8 to 21.7) | 19.5 (16.7 to 21.9) |
| ESV (%) | 20.5 (16.6 to 23.9) | 20.0 (16.8 to 23.3) | 21.4 (17.2 to 25.5) | 17.0 (13.6 to 20.2) | 16.8 (13.0 to 20.4) | 18.9 (15.7 to 22.1) |
| LVEF (%) | 14.5 (10.1 to 19.6) | 13.7 (11.3 to 16.3) | 18.0 (14.9 to 21.2) | 9.5 (7.5 to 11.4) | 13.3 (9.7 to 16.8) | 13.3 (9.8 to 17.4) |
| GLS (%) | 23.5 (17.0 to 31.1) | 26.3 (19.4 to 33.6) | 21.3 (14.9 to 27.8) | 17.2 (14.4 to 20.0) | 15.7 (12.5 to 19.0) | 21.0 (17.3 to 24.8) |
| **Bland Altman Limits of Agreement (95% CI)** | | | | | | |
| EDV (ml) | -52.5, 22.4 | -44.2, 20.5 | 5.9, 61.2 | -36.7, 37.6 | -15.2, 52.3 | -7.1, 48.6 |
| ESV (ml) | -26.5, 27.1 | -20.5, 23.5 | -16.6, 26.7 | -21.2, 20.1 | -14.7, 25.3 | -13.4, 22.9 |
| LVEF (%) | -23.2, 11.1 | -24.1, 12.6 | -10.0, 29.5 | -12.8, 14.1 | -13.0, 20.4 | -10.9, 21.4 |
| GLS (%) | -10.1, 7.0 | -10.6, 2.9 | -7.0, 8.3 | -8.9, 4.7 | -7.3, 5.7 | -9.6, 3.6 |

E1 -4 = Expert 1 – 4, EDV = End diastolic volume, ESV = End systolic volume, GLS = Global longitudinal strain, LVEF = Left ventricular ejection fraction

**Supplemental Table 11: [Individual Expert Precision Metrics**. Individual expert precision]

|  | **E1** | **E2** | **E3** | **E4** |
| --- | --- | --- | --- | --- |
| **Mean Absolute Difference** | | | | |
| EDV (ml) | 11.5 (9.3 to 14.3) | 11.3 (8.7 to 14.0) | 11.5 (9.3 to 14.0) | 9.3 (7.6 to 11.3) |
| ESV (ml) | 7.8 (6.1 to 9.9) | 7.0 (5.4 to 8.7) | 6.6 (5.4 to 7.8) | 6.9 (5.4 to 8.5) |
| LVEF (%) | 7.1 (5.7 to 8.8) | 6.6 (5.4 to 7.8) | 4.5 (3.8 to 5.3) | 8.1 (6.5 to 9.8) |
| GLS (%) | 2.6 (2.1 to 3.0) | 3.3 (2.6 to 4.0) | 1.5 (1.1 to 1.8) | 2.7 (2.2 to 3.3) |
| **Minimal Detectable Change** | | | | |
| EDV (ml) | 14.9 (10.5 to 19.5) | 15.2 (10.0 to 19.8) | 13.0 (9.5 to 16.3) | 11.5 (8.2 to 14.8) |
| ESV (ml) | 10.4 (6.7 to 14.7) | 9.3 (6.1 to 12.5) | 7.4 (5.2 to 9.2) | 8.9 (5.7 to 12.3) |
| LVEF (%) | 9.2 (6.3 to 12.4) | 8.1 (6.1 to 10.1) | 5.3 (4.0 to 6.6) | 10.1 (7.3 to 12.8) |
| GLS (%) | 3.0 (2.3 to 3.7) | 4.0 (2.9 to 5.2) | 1.8 (1.3 to 2.3) | 3.3 (2.4 to 4.1) |
| **Within Subject Co-Efficient of Variation** | | | | |
| EDV (%) | 9.9 (7.9 to 11.8) | 11.7 (9.0 to 14.6) | 11.5 (9.0 to 13.8) | 11.2 (8.6 to 13.8) |
| ESV (%) | 18.3 (14.4 to 22.3) | 15.2 (11.7 to 19.5) | 13.7 (11.5 to 15.9) | 18.4 (14.2 to 22.5) |
| LVEF (%) | 11.7 (8.5 to 14.8) | 11.2 (9.3 to 13.0) | 6.6 (5.6 to 7.5) | 14.5 (11.7 to 17.2) |
| GLS (%) | 18.9 (13.8 to 24.6) | 19.2 (15.1 to 23.4) | 7.4 (5.9 to 8.7) | 16.6 (13.6 to 19.5) |
| **Bland-Altman Limits of Agreement** | | | | |
| EDV (ml) | -31.3, 29.1 | -31.3, 29.7 | -32.3, 20.7 | -23.5, 23.1 |
| ESV (ml) | -20.6, 22.2 | -18.7, 18.9 | -17.7, 12.4 | -18.5, 17.7 |
| LVEF (%) | -19.5, 17.8 | -16.4, 16.1 | -10.5, 10.8 | -19.4, 21.2 |
| GLS (%) | -5.2, 6.9 | -7.6, 8.8 | -3.9, 3.4 | -6.7, 6.7 |

E1 -4 = Expert 1 – 4, EDV = End diastolic volume, ESV = End systolic volume, GLS = Global longitudinal strain, LVEF = Left ventricular ejection fraction

**Supplemental Table 12: [Analysis Feasibility.** Feasibility of LVEF and GLS measurement by analysis method]

| **n=60** | **AI Analysis** | **Expert Analysis** |
| --- | --- | --- |
| **SBP LVEF** | 97% | 97% |
| **3DE LVEF** | - | 74% |
| **GLS** | 96% | 92% |

3DE =3-dimensional echocardiography, AI = Artificial intelligence. GLS = Global longitudinal strain, LVEF = Left ventricular ejection fraction. SBP = Simpsons Biplane

**Supplemental Table 13: [Clinical Trial Sample Size Effects.** Effects of AI precision on predicted study sample size to appropriately detect a given change in LVEF or GLS]

|  | **LVEF** | | **GLS** |
| --- | --- | --- | --- |
|  | **Relative sample size for 5% Change**  **(n = 60)** | **Relative sample size for 3% Change**  **(n = 166)** | **Relative sample size for 3% Change**  **(n=23)** |
| **AI vs expert analysis** | 33% | 31% | 51% |

AI = Artificial intelligence. GLS = Global longitudinal strain, LVEF = Left ventricular ejection fraction

**Supplementary Figures 1. [CONSORT style diagram.** AI = Artificial Intelligence]

**Supplementary Figures 2. [Regression Analysis Plots.** Scatter plots of the regression analysis of AI vs pooled expert analysis for LVEF, LVEDV, LVESV and GLS, AI = β₀ + β₁·Expert. LVEF Scatter plots of the regression analysis of individual expert vs remaining pooled experts for comparison. AI = Artificial Intelligence, E1 – E4 = Expert 1 – 4, EDV = End diastolic volume, EF = Left ventricular ejection fraction, ESV = End systolic volume, GLS = Global longitudinal strain]

**Supplementary Figures 3. [CMR Agreement.** A) Inter-observer LVEF MAD compared to expert CMR results. B) Inter-observer LVEF WSCoV compared to expert CMR results. C) Inter-observer LVEF difference to expert CMR result. AI = Artificial Intelligence, E1 – E4 = Expert 1 – 4, EF = Left ventricular ejection fraction, MAD = Mean Absolute Difference, WSCoV = Within Subject of Co-Efficient of variation]

**Supplementary Figures 4: [Bland-Altman Plots.** Individual reader Bland-Altman plot for test-retest studies. AI = Artificial Intelligence, E1 – E4 = Expert 1 – 4, EDV = End diastolic volume, EF = Ejection fraction ESV = End systolic volume, GLS = Global longitudinal strain]

**Supplementary Figure 5. [Clinically significant test-retest differences.** Bar plots of significant test-retest differences, AI = Artificial Intelligence, E1 – E4 = Expert 1 – 4, GLS = Global longitudinal strain, LVEF = Left ventricular ejection fraction, TRT = Test-Retest]

**Supplementary Figures 6. [Intra-observer test-retest CTRCD Diagnosis.** Bar plots of ESC defined CTRCD across test-retest studies by analysis method. AI = Artificial Intelligence, CTRCD = Cancer therapy related cardiac dysfunction, E1 – E4 = Expert 1 – 4, EDV = End diastolic volume, ESV = End systolic volume, GLS = Global longitudinal strain, LVEF = Left ventricular ejection fraction, TRT = Test-Retest]

**Supplementary Figure 7. [Stratified measurement precision.** A) LVEF MAD across participant strata. B) GLS MAD across participant strata. GLS = Global longitudinal strain, LVEF = Left ventricular ejection fraction, MAD = Mean absolute difference.

**Blank page**
